# The complete chloroplast genome of *Sargassum fusiforme*

**DOI:** 10.1080/23802359.2019.1710296

**Published:** 2020-01-14

**Authors:** Tao Liu, Yutong Cui, Xuli Jia, Binbin Chen, Zengling Ma, Huixi Zou, Shengqin Wang, Mingjiang Wu

**Affiliations:** a College of Life Sciences, Yantai University, Yantai, Shandong, China;; b College of Life and Environmental Science, Wenzhou University, Wenzhou, Zhejiang, China;; c College of Marine Life Sciences, Ocean University of China, Qingdao, Shandong, China

**Keywords:** *Sargassum fusiforme*, phylogenetic analysis, chloroplast genome

## Abstract

The complete chloroplast genome sequence of *Sargassum fusiforme* is presented here. Circular mapping revealed that the complete chloroplast DNA sequence of *S. fusiforme* was 124,298 bp in length and had an overall AT content of 69.57%, including 137 protein-coding genes, 2 open reading frames, 28 transfer RNA genes, and 6 ribosomal RNA genes. The phylogenetic tree based on Bayesian shows that all kinds of Phaeophyceae were clustered into two monophyletic groups.

*Sargassum fusiforme* is a kind of brown algae that belongs to the family of Sargassaceae (Dixon et al. 2014). It is one of the most widely consumed seaweeds, grows on lower intertidal and upper subtidal rocks, primarily distributed in the temperate seaside areas of the northwest Pacific Ocean, including China, Japan, and Korea (Yu et al. 2012; Hu et al. 2013). *Sargassum fusiforme* is not only as food but also as traditional Chinese herbal medicine. Some studies have confirmed that the main constituents of *S. fusiforme* are polysaccharides, together with amino acids, sterols, and microelements (Liu et al. 2012). It is one of the most important seaweeds for mariculture in China.

Here, we report the complete chloroplast genome sequence of *S. fusiforme* to provide a genomic resource and to clarify the phylogenetic relationship of this seaweed with other species in the Phaeophyceae class. The specimen was collected from Wenzhou, Zhejiang Province, China (N27°50′25.19″, E121°01′23.36″), and stored at the Culture Collection of Seaweed at the Ocean University of China with an accession number 2015040102. Total DNA was extracted using the modified CTAB method (Doyle and Doyle 1990). Paired-end reads were sequenced using Illumina HiSeq × Ten system (Illumina, San Diego, CA). The complete chloroplast genomes were assembled using the program NOVOPlasty (Dierckxsens et al. 2017) with its close relative *Sargassum horneri* as the reference (GenBank accession number: MN265366). Sequence annotation was added using Geneious Prime. At last, the annotated sequence was submitted to GenBank with the accession number MN794016.

The complete chloroplast genome of *S. fusiforme* is a typical circular structure of 124,298 bp in total length with the overall A + T content of 69.57%. The nucleotide composition was 34.73% A (43,167 bp), 15.56% G (19,346 bp), 34.84% T (43,302 bp), 14.87% C (18,483 bp). There are a total of 173 genes in the chloroplast genome, comprising 137 protein-coding genes, 2 open reading frames, 28 transfer RNA genes, and 6 ribosomal RNA genes. All genes show the typical gene arrangement conforming to the Phaeosporeae consensus. Only 2 genes (*psbF* and *rpl3*) use the typical GTG as the start codon, the remaining protein-coding genes treated the typical initiation ATG as the start codon. About 78.42% protein-coding genes had TAA as termination codon and the remainings were TAG (16.55%) and TGA (5.03%). All transfer RNA genes have the typical cloverleaf structure.

All Phaeophyceae species with complete chloroplast genome available in the NCBI were selected to construct the phylogenetic tree by the Bayesian method, with *Caulerpa lentillifera* (GenBank accession number MN201587) which was sequenced and published by ourselves served as outgroup. The phylogenetic tree based on combined 34 protein-encoding genes exhibited that all species of Phaeophyceae were clustered into two monophyletic groups. *Sargassum fusiforme* firstly groups with the species of Fucales constituting a monophyletic clade (Figure 1). Results support current taxonomic systems (Dixon et al. 2014).

**Figure 1.**
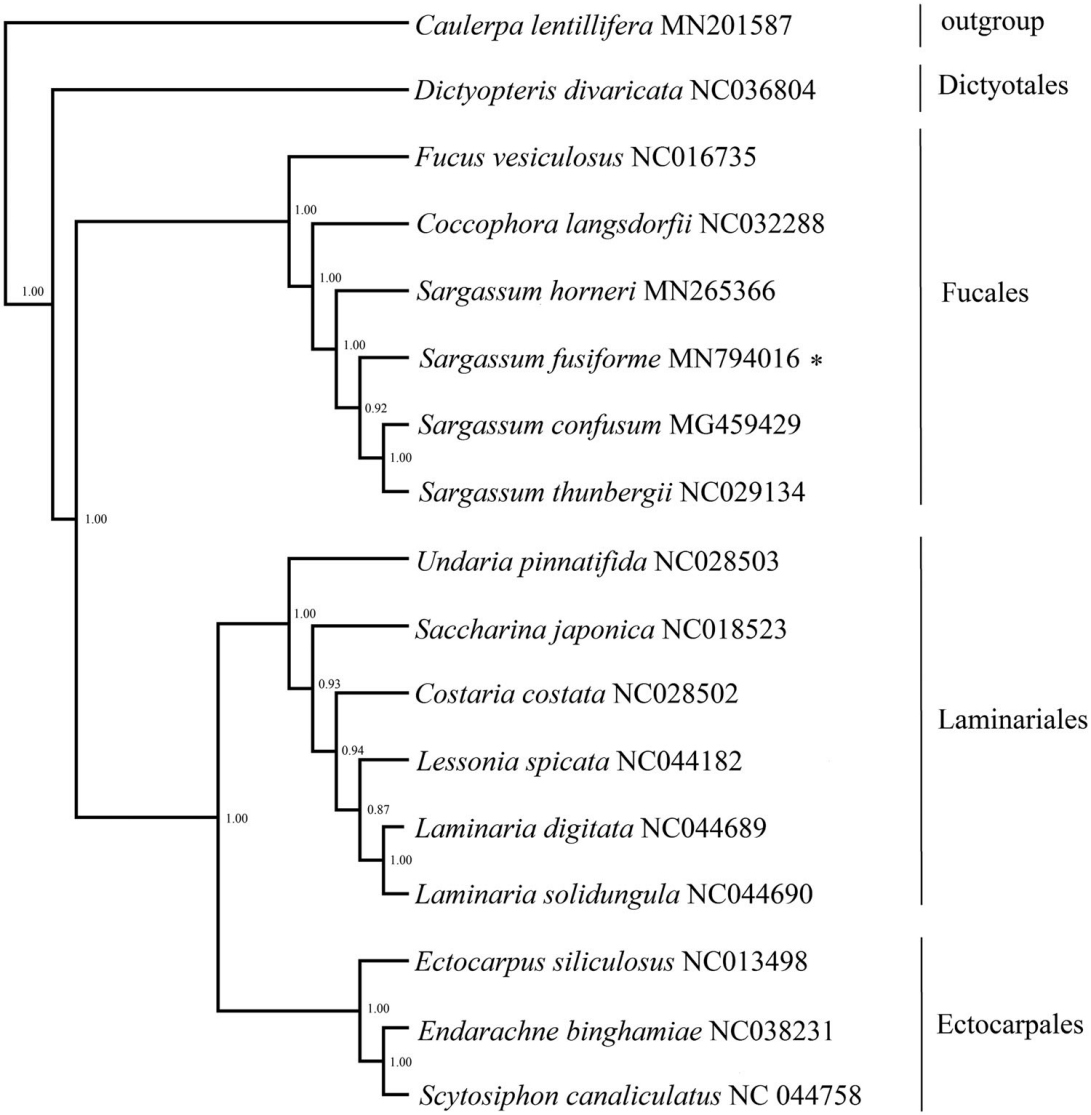
Phylogenetic tree based on complete chloroplast genome of Phaeophyceae. Support values for each node were calculated from Bayesian posterior probability (BPP). Asterisks following species names indicate newly determined chloroplast genomes.
